# Clinical-scale manufacturing of CD137+ enriched tumor-reactive T cells for glioblastoma in the CliniMACS Prodigy

**DOI:** 10.3389/fimmu.2026.1866645

**Published:** 2026-10-12

**Authors:** Arina Riabinska, Elvira Criado-Moronati, Emre Balta, Denis Frison, Cristina Berganza-Irusqueta, Tümay Telatar, Donata Maciaczyk, Johanna Engeln, Laura Fischer, Marieke Domdey, Imran Siddiqui, Velita Miranda, Teresa Schmidt, Sied Kebir, Yahya Ahmadipour, Martin Glas, Ulrich Herrlinger, Matthias Schneider, Anna-Laura Potthoff, Marcus Czabanka, Thomas Broggini, Sofia Stenqvist, Lisa M. Nilsson, Jonas A. Nilsson, Martina Ott, Andrzej Dzionek, Ian R. Hardy

**Affiliations:** 1 Miltenyi Biotec GmbH, Bergisch Gladbach, Germany; 2 Division of Clinical Neurooncology, University Hospital of Essen, Essen, Germany; 3 Department of Neurology and Center for Translational Neuro- and Behavioral Sciences (C-TNBS), University Hospital of Essen, Essen, Germany; 4 Department of Neurosurgery and Spine Surgery, Center for Translational Neuro- and Behavioural Sciences (C-TNBS), University Hospital of Essen, Essen, Germany; 5 Department of Neurology and Neurooncology, St. Marien Hospital, Lünen, Germany; 6 Department of Neurooncology, Center for Neurology, University Hospital Bonn, Bonn, Germany; 7 Department of Neurosurgery, University Hospital Bonn, Bonn, Germany; 8 Department of Neurosurgery, University Hospital Frankfurt, Frankfurt, Germany; 9 Department of Surgery, Institute of Clinical Sciences, University of, Gothenburg, Sweden

**Keywords:** CD137+ T cells, CliniMACS Prodigy^®^, glioblastoma, T cell therapy, tumor infiltrating lymphocyte (TIL)

## Abstract

**Background:**

Most tumor-infiltrating lymphocyte (TIL) studies and therapies have focused on tumors with high T-cell infiltration and mutational burden. Glioblastoma (GBM) represents a challenging indication for TIL therapy because of its limited T-cell infiltration and low mutational burden. We investigated whether targeted enrichment of tumor-reactive T cells using CD137-based selection could enable robust, clinical-scale production of TIL products from GBM.

**Methods:**

Ultrasonic aspirates (UAs), routinely collected during GBM resections, were evaluated as a source of TILs for clinical manufacturing. TILs were generated from GBM UAs under GMP-like conditions at clinical scale using the closed, automated TRT Process on the CliniMACS Prodigy. Matched TRT products that were enriched for CD137+ cells, depleted of CD137+ cells, or left unselected were generated in parallel at full manufacturing scale. Products were characterized by cellular and transcriptomic analyses and evaluated in functional assays for cytotoxicity against autologous and non-autologous patient-derived GBM cell lines.

**Results:**

Cellular and transcriptomic analyses revealed that UA-derived CD137+ (4-1BB/TNFRSF9) T cells were clonally expanded, exhibited activated and memory phenotypes, and displayed transcriptional profiles associated with tumor reactivity. The closed manufacturing process preserved phenotypic features previously associated with clinical benefit in other TIL indications. Functional assays demonstrated potent, tumor-reactive cytotoxicity of CD137-enriched TRT products against autologous GBM cell lines compared with TILs generated in parallel without selection.

**Discussion/Conclusions:**

These findings establish the feasibility of a clinical-scale TIL manufacturing approach using the CliniMACS Prodigy that enables production of clinically relevant quantities of TILs from GBM while enriching for cells with tumor-reactive characteristics. This approach may broaden the application of TIL therapy to immunologically cold tumors with limited T-cell infiltration or low mutational burden that have historically been challenging to manufacture using conventional TIL production methods.

## 1 Introduction

Tumor-infiltrating lymphocyte (TIL) adoptive cell therapy has demonstrated promising clinical activity across several solid tumors, with favorable safety and durable responses (1, 2). These advances have led to regulatory approval of TIL therapy for melanoma, including FDA approval of AMTAGVI™ and the reimbursement of hospital-based TIL therapy in the Netherlands (3, 4). Despite successes in several inflamed tumor types, translation of TIL therapy to immunologically “cold” tumors remains challenging. Cold tumors are characterized by low mutational burden, limited T cell infiltration, pronounced immunosuppression, and limited tissue availability, all of which challenge conventional TIL manufacturing approaches and therapeutic efficacy (5–8).

Conventional TIL manufacturing typically begins with generation of tumor fragments or enzymatic digestion of the tumor, followed by an initial expansion phase (pre-REP) designed to promote outgrowth of endogenous TILs. This process commonly relies on high-dose IL-2 stimulation and can be highly variable, with some tumor specimens failing to generate sufficient numbers of T cells for subsequent manufacturing. Following successful initial outgrowth, a rapid expansion protocol (REP), typically using feeder cells and additional stimulation, is employed to achieve the large-scale expansion required for clinical administration. Although effective in highly infiltrated tumors, this multistep process presents challenges for poorly inflamed tumors, where rare tumor-reactive T cells may be diluted by non-reactive populations, fail to expand, or be lost during early culture. Furthermore, the reliance on variable tumor fragment outgrowth and operator-dependent culture steps has limited standardization and automation of conventional TIL manufacturing.

To address these limitations, we previously developed a TIL manufacturing strategy using the CliniMACS Prodigy Tumor Reactive T (TRT) cell Process, enabling enrichment of tumor-reactive T cells based on CD137 expression (4-1BB/TNFRSF9) (9). This approach allows direct enrichment of tumor-reactive T cells from tumor digests, bypassing conventional TIL fragment outgrowth and enabling expansion from limited starting cell numbers using an automated rapid expansion protocol (REP). Here, we extend this strategy to glioblastoma (GBM), a highly heterogeneous and poorly infiltrated tumor that has historically been difficult to target with adoptive cell therapies (10, 11).

Recent studies using chimeric antigen receptor (CAR) T cells in GBM have demonstrated biological activity, but antigen loss remains a significant barrier (10, 12–15). In contrast, the polyclonal and diverse TCR repertoire inherent to TIL therapy offers the potential to address tumor heterogeneity. Early clinical experience with GBM-derived TILs, including a small trial conducted in 1999 and a more recent single case report in 2025, suggested feasibility and preliminary evidence of clinical activity, but broader development has been limited by difficulties in generating sufficient numbers of T cells from small, poorly infiltrated tumor specimens (16–20).

To establish a robust, GMP-compliant manufacturing process, we utilized a workflow in which ultrasonic aspirates (UA) material collected during GBM surgeries served as the input for CD137-based enrichment within the closed and automated TRT Process. Previous studies have shown that UA material is comparable to conventional tumor fragments while offering improved cell yield and accessibility (21). Across multiple patients, CD137-enriched TIL products were consistently manufactured and exhibited phenotypic and functional characteristics associated with favorable TIL therapy outcomes, including high viability, a favorable T cell subset balance, and limited expression of exhaustion markers. These findings support the feasibility of generating clinically relevant TIL products from GBM and highlight the potential of combining CD137-based selection, ultrasonic aspirate-derived tumor material, and automated manufacturing to enable adoptive cell therapy for immunologically cold tumors.

## 2 Results

### 2.1 Surgical ultrasonic aspirates from GBM are a reliable source of TILs

In our previous work, we demonstrated successful generation of tumor-reactive T cells (TRTs) from melanoma and other tumor types using CD137 as an antigen-agnostic marker of tumor reactivity with the TRT Process on the CliniMACS Prodigy, a closed and GMP-compliant cell manufacturing system (9). To investigate whether this methodology could be applied to the isolation and manufacturing of TRTs from GBM, a notably immunologically cold tumor with low T cell infiltration, we established a workflow using the TRT Process (**Figure 1A**).

**Figure 1:**
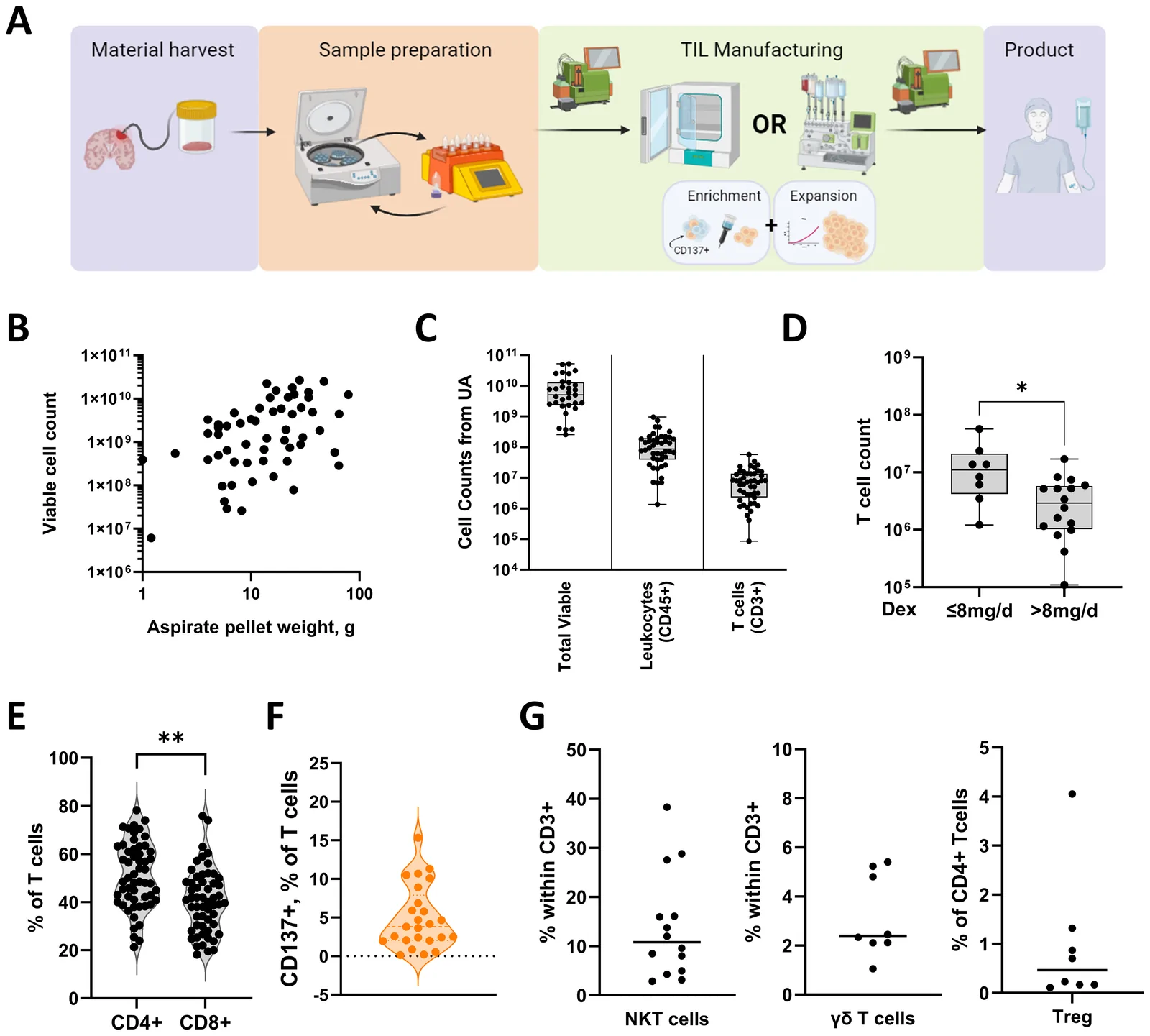
Surgical ultrasonic aspirates (UA) from GBM are a reliable source of TILS. **(A)** Schematic of the workflow for generating tumor-reactive T cells (TRTs) from GBM ultrasonic aspirates (UAs) using the CliniMACS Prodigy TRT Process. **(B)** Weight of surgical UAs and total viable cells recovered following tissue dissociation (n = 30). **(C)** Total viables, leukocyte (CD45+), and T cell (CD3+) counts recovered from GBM UA digests. **(D)** T cell counts in GBM UA digests from patients receiving ≤8 or >8 mg/day dexamethasone before surgery. **(E)** Frequency of CD4+ and CD8+ T cells among total T cells in GBM UA digests (n=56). **(F)** Frequency of CD137+ TILs in freshly digested GBM UA before overnight culture (n=27). **(G)** Frequency of NKT-like (CD3+/CD56+) γδ T cells (γδTCR+) among total T cells, and frequency of regulatory T cells (CD25^high^/CD127-/FOXP3+) among CD4+ T cells within freshly digested GBM UA.

A total of 65 ultrasonic aspirates from IDH-wildtype GBM patients were collected during surgery and prepared for single cell analysis. Aspirate weight, pellet weight and total viable cell number within UA varied considerably between donors (**Figure 1B**, **Supplementary Figure S1A**). As expected, the majority (~88%) of the viable cells within the digest were negative for CD45 and CD31 and exhibited scatters consistent with neuronal or glioblastoma cells (**Supplementary Figures S1B, C**). The remaining cellular fraction of the UA consisted of CD45+ leukocytes (~11%) and CD31+ endothelial cells (~1%) (**Supplementary Figure S1C**). Within the leukocyte compartment, the majority of the cells were CD11b+ myeloid cells (65%) with the remainder being comprised of CD3+ T cells (~13%), CD56+ NK cells (~4%) and CD20+ B cells (<1%) of the total leukocyte population (**Supplementary Figure S1C**). Among the CD45+/CD11b+ myeloid compartment, we identified CD66b+ tumor-associated neutrophils (TANs), CD66b- tumor-associated macrophages (TAMs), microglia, monocyte derived macrophages (MDMs), and myeloid derived suppressor cells (MDSCs) (**Supplementary Figure S1C**). These results are in accordance with previous descriptions of GBM UA material (21, 22). Despite substantial variability in the cellularity among patients, GBM UA contained a mean of 1.1x10^10^ viable cells, including ~1.5x10^8^ CD45+ leukocytes and yielded an average of 9.7x10^6^ CD3+ T cells. Notably, 61 out of 65 (94%) surgical samples yielding at least 1x10^6^ T cells (**Figure 1C**). This observation indicated that surgical aspirates from GBM patients represent a robust and feasible starting material for clinical-scale TIL manufacturing. We further observed a reduced TIL yield in UA from patients receiving the anti-inflammatory corticosteroid dexamethasone at doses >8mg/d (**Figure 1D**, **Supplementary Figure S2A**, **Supplementary Table 1**). Consistent with reports linking dexamethasone use in GBM with impaired responses to immune checkpoint blockade, we also observed a trend towards a more differentiated T cell phenotype in samples from patients receiving higher doses of dexamethasone treatment before surgery (**Supplementary Figure S2B**) (23). Notably, no significant differences in expression of activation or exhaustion markers, including CD137, were detected between dexamethasone treatment groups (**Supplementary Figures S2C-E**). The CD4/CD8 ratios among TILs were highly variable between patients, with an overall skewing toward the CD4 compartment (**Figure 1E**). Importantly, an average of 5% of T cells expressed CD137 (**Figure 1F**), a costimulatory receptor transiently induced following TCR-mediated activation. CD137 has been used by our group and others as an antigen-agnostic marker to enrich tumor-reactive T cells across multiple tumor indications (24, 25). Additional lymphocytic populations were also noted, including of CD3+CD56+ NKT-like cells (11%), γδ T cells (2.4% among CD3+) and regulatory T cells (Tregs) (1% of CD4+ T cells) (**Figure 1G**).

### 2.2 GBM CD137+ TILs possess phenotypic and transcriptional characteristics associated with therapeutic potential

Previous work from our team and others has demonstrated that selection of CD137+ TIL from tumor digests can significantly enrich antitumor reactivity in TIL products. We therefore characterized the CD137+ TIL population within the GBM UA. CD137+ TILs expressed higher levels of activation and exhaustion markers, including PD1 and TIGIT, as well as CD39 and CD103, relative to CD137- TILs (**Figure 2A**). Most activation and exhaustion markers, with the exception of CD103, did not differ between CD4+ and CD8+ T cell subsets (**Supplementary Figures S3C-H**). Interestingly, despite exhibiting a more activated/exhausted phenotype, CD137+ TIL displayed a less differentiated T cell subset profile, with enrichment of T stem cell memory (Tscm) cells and reduced frequencies of T effector (Teff) subpopulations. Relative frequencies of naïve T cells (Tn), central memory (Tcm) and effector memory T cell (Tem) subsets were not significantly different between CD137+ and CD137- TILs (**Figure 2B**, **Supplementary Figure S3A, E, F**). Expression of CD27 and CD28 in TILs is associated with a less differentiated T cell state and has been reported to correlate with improved therapeutic outcomes (1, 26). Approximately 38% of CD137+ TILs co-expressed CD27 and CD28, indicating a phenotype associated with favorable therapeutic potential (**Figure 2C**, **Supplementary Figure S3G, H**).

**Figure 2:**
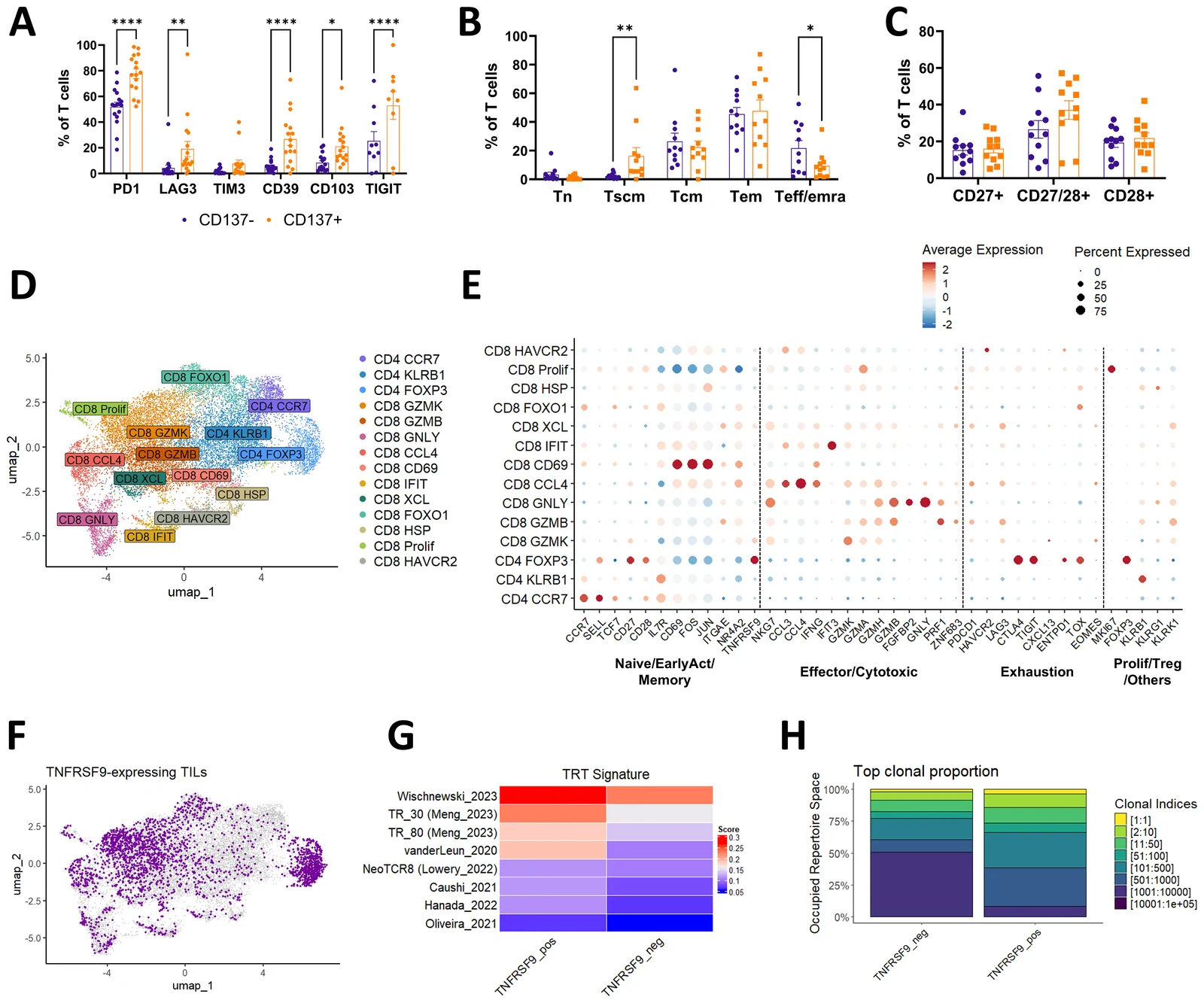
GBM CD137+ TILs possess phenotypic and transcriptional characteristics associated with therapeutic potential. **(A)** Expression of activation and exhaustion markers in CD137- (purple) and CD137+ (orange) CD3+ TILs in GBM UA digests (n = 16) **(B)** Distribution of T cell memory subsets of CD137- (orange) and CD137+ (purple) TILs (n = 11) Memory subset gating strategy described in **Supplementary Figure 3A**. **(C)** Co-expression of CD27 and CD28 on CD137- and CD137+ TILs (n=11). **(D)** UMAP visualization of T cell clusters identified by single-cell RNA sequencing of GBM UA-derived TILs (n = 6) **(E)** Expression of genes selected genes associated with distinct T cell across the identified clusters (n = 6) **(F)** Distribution of TNFRSF9-expressing (CD137+) T cells within the single cell dataset. **(G)** Tumor-reactive T cell (TRT) signature scores of TNFRSF9-expressing and TNFRSF9-non-expressing CD8+ T cells based on eight published tumor-reactive T cell gene signatures. **(H)** TCR clonal repertoire divided into indices (e.g., top 1, top 2-10, etc) among TNFRSF9-expressing and TNFRSF9-non-expressing T cells.

To gain a deeper understanding of the T cell subset composition within UA, we performed single-cell RNA sequencing of six GBM TIL samples. Unsupervised clustering of 15,819 T cells resulted in 14 distinct clusters, which were annotated based on their transcriptional profiles. These included three CD4+ and eleven CD8+ TIL clusters (**Figure 2D**, **Supplementary Figure S2F**). We identified four CD8 clusters with an activated phenotype, characterized by expression of granzymes (*GZMK*, *GZMB*) and effector molecules (*GNLY*, *CCL4*, *NKG7*). Recently, Wang et al. described a clonally expanded *GZMK*+ effector CD8+ population in GBM with potential antitumor activity (27). In our dataset, we similarly observed a *GZMK*+ CD8+ population that exhibited low expression levels of exhaustion genes (*PDCD1*, *HAVCR2*, *LAG3*) and contained a subset of cells expressing *CXCL13* and *TNFRSF9* (encoding CD137) genes previously associated with tumor reactivity (**Figure 2E**). Expression of *TNFRSF9* was present across several clusters, particularly CD8 GZMK and GZMB clusters, as well as in *FOXP3*+ CD4 Treg cells (**Figure 2F**) suggesting that CD137-expressing T cells are represented within several transcriptional profiles.

We further independently analyzed our single-cell transcriptomic data set using eight previously published tumor-reactivity signatures (28–34). *TNFRSF9*-expressing T cells had increased tumor-reactivity scores relative to their negative counterparts (**Figure 2G**). TCR clonotype analysis also revealed that *TNFRSF9* positive T cells predominantly consisted of large and medium-sized clones, in contrast to *TNFRSF9* negative T cells, which were enriched for smaller clones (**Figure 2H**). Taken together, these data suggest the presence of clonally expanded, tumor-reactive CD137+ T cell populations within the UA obtained during GBM resection, supporting their potential as a clinically relevant starting material for TIL manufacturing.

### 2.3 Tumor-reactive TILs can be enriched from GBM surgical aspirates and expanded with the CliniMACS prodigy TRT process

Given the consistent presence of CD137+ TILs in GBM tumor digest, with surface marker and transcriptional profiles indicative of tumor reactivity, we sought to evaluate this material as a starting source for TIL manufacturing with the CliniMACS Prodigy TRT process. Overnight culture of the GBM tumor digests resulted in a significant increase in the frequency of CD137+ TILs in most patient material from both the bench-top small scale and the GMP-compliant CliniMACS Prodigy (**Figure 3A**). After overnight culture in the CliniMACS Prodigy, CD137+ cells were labeled and magnetically enriched. Recovered T cells were over 90% CD137+ (**Figure 3B**). For process comparison and later downstream analysis, we also expanded CD137-depleted TIL and unenriched TILs from the same patient on parallel CliniMACS Prodigy devices. Across 11 independent GMP-scale manufacturing runs, CD137+ TRT products were successfully generated in 10 cases (91% manufacturing success rate). The single manufacturing failure was attributed to incomplete irradiation of feeder cells used during expansion, resulting in feeder cell outgrowth and subsequent contamination. Following optimization of the feeder cell preparation procedure, no further manufacturing failures were observed (**Supplementary Table 2**). High fold expansions were observed from the CD137+ TRT, CD137- depleted TIL and the unenriched TIL products, resulting in highly viable and consistent final products (**Figures 3C–F**). Interestingly, overall fold expansion and viability was higher using the CliniMACS Prodigy TRT Process compared to the smaller scale manual culture (**Supplementary Figure S4**).

**Figure 3:**
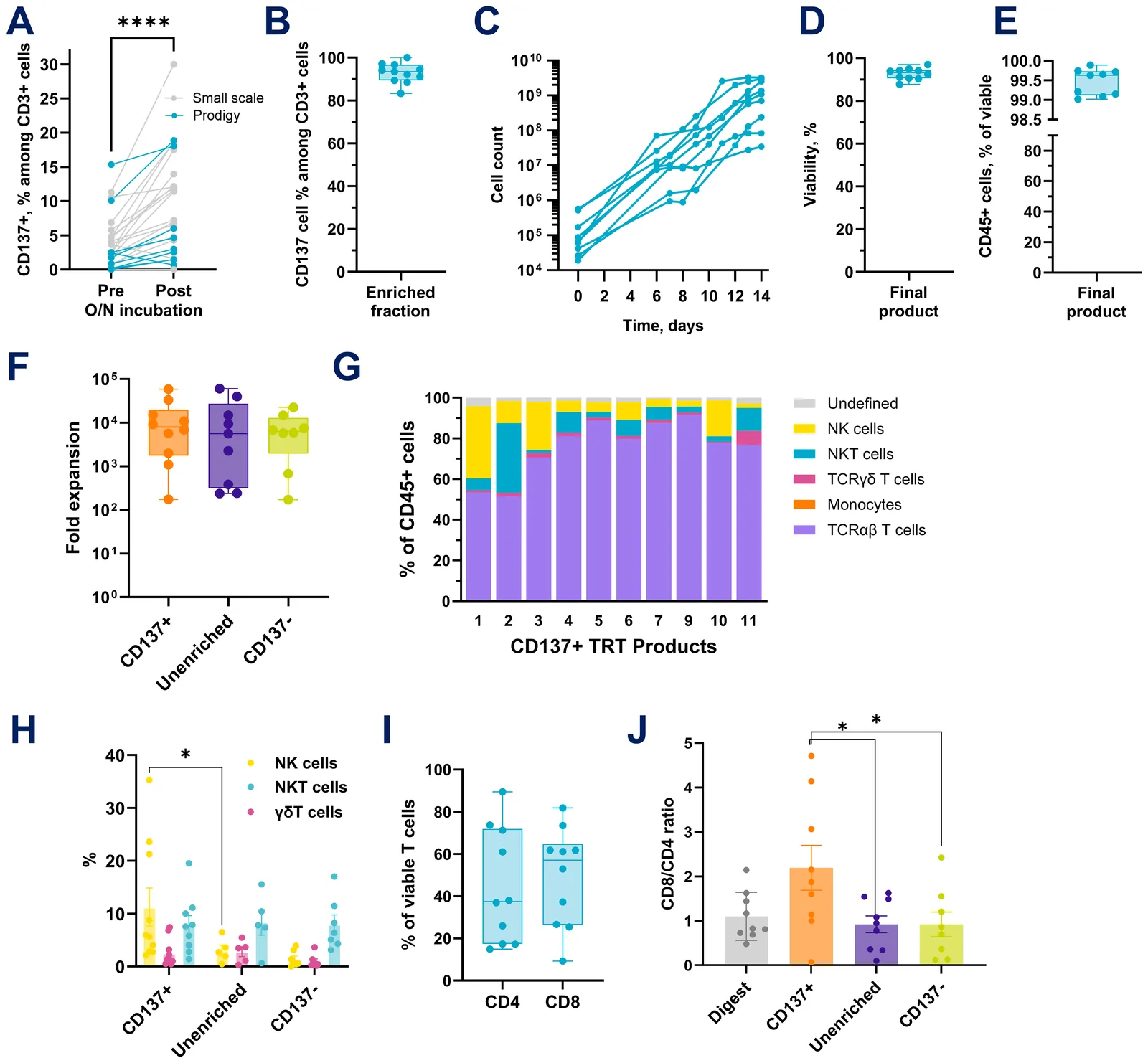
Tumor-reactive TILs can be enriched from GBM surgical aspirates and expanded with the CliniMACS Prodigy TRT Process. **(A)** The fraction of T cells within digested UA expressing CD137 was assessed before and after the overnight culture. **(B)** Frequency of CD137+ T cells after magnetic enrichment for CD137 on the CliniMACS Prodigy. **(C)** Ennumeration of GBM-TIL during the TRT Process. At the end of culture CD137+ TRT cells were analyzed for **(D)** viability; **(E)** and fraction of CD45+ cells. **(F)** No significant difference in fold expansion was observed between CD137+ TRT, unenriched TIL and CD137-depleted TIL products. **(G)** Cellular distribution of the CD137+ TRT products. The product numbers are given according to **Supplementary Table 2**. **(H)** Fractions of NK (CD3- CD56+), NKT-like (CD3+ CD56+) and yδT cells (CD3+ γδTCR+) within CD137+ TRT, unenriched or CD137-depleted TIL products. **(I)** CD4 and CD8 T cell fractions in the CD137+ TRT products. **(J)** CD8 to CD4 ratios in starting tumor digest and products generated from within CD137+ TRT, unselected TIL, or CD137-depleted TIL products.

CD137+ TRT products were predominantly composed of CD3+ αβT cells, with a variable population NK and NKT-like cells observed across donors (**Figure 3G**). NK cells were slightly more prevalent in the CD137+ TRT products, relative to the unenriched TIL products (**Figure 3H**). The CD137+ TRT products also had an increased CD8/CD4 ratio compared to unenriched TIL, a parameter associated with favorable TIL therapy outcomes (**Figures 3I, J**) (26, 35). Because CD137 is highly expressed in Tregs in the GBM tumor digest, we assessed whether this population expanded during manufacturing and was retained in the final CD137+ TRT product. However, Tregs were rarely detected (~0.65%) in the final CD137+ TRT product (**Supplementary Figure S4G**).

### 2.4 CD137+ TRT products maintain favorable phenotypic and transcriptional features associated with therapeutic potential

CD137+ TRT products generated using the CliniMACS Prodigy TRT Process consisted predominantly of central memory (Tcm) T cells (~80%), with the remaining cells displaying an effector memory (Tem) phenotype (**Figure 4A**). This distribution appeared to be influenced by the choice of manufacturing platform as CD137+ TRTs generated by the small-scale benchtop process displayed a more effector-memory dominated subset profile (**Supplementary Figure S5A**). The T cell subset distribution was similar in CD137+ TRT, CD137-depleted TIL and unenriched TIL products, suggesting that automated expansion may facilitate the maintenance of Tcm cells (**Supplementary Figure S5A**).

**Figure 4:**
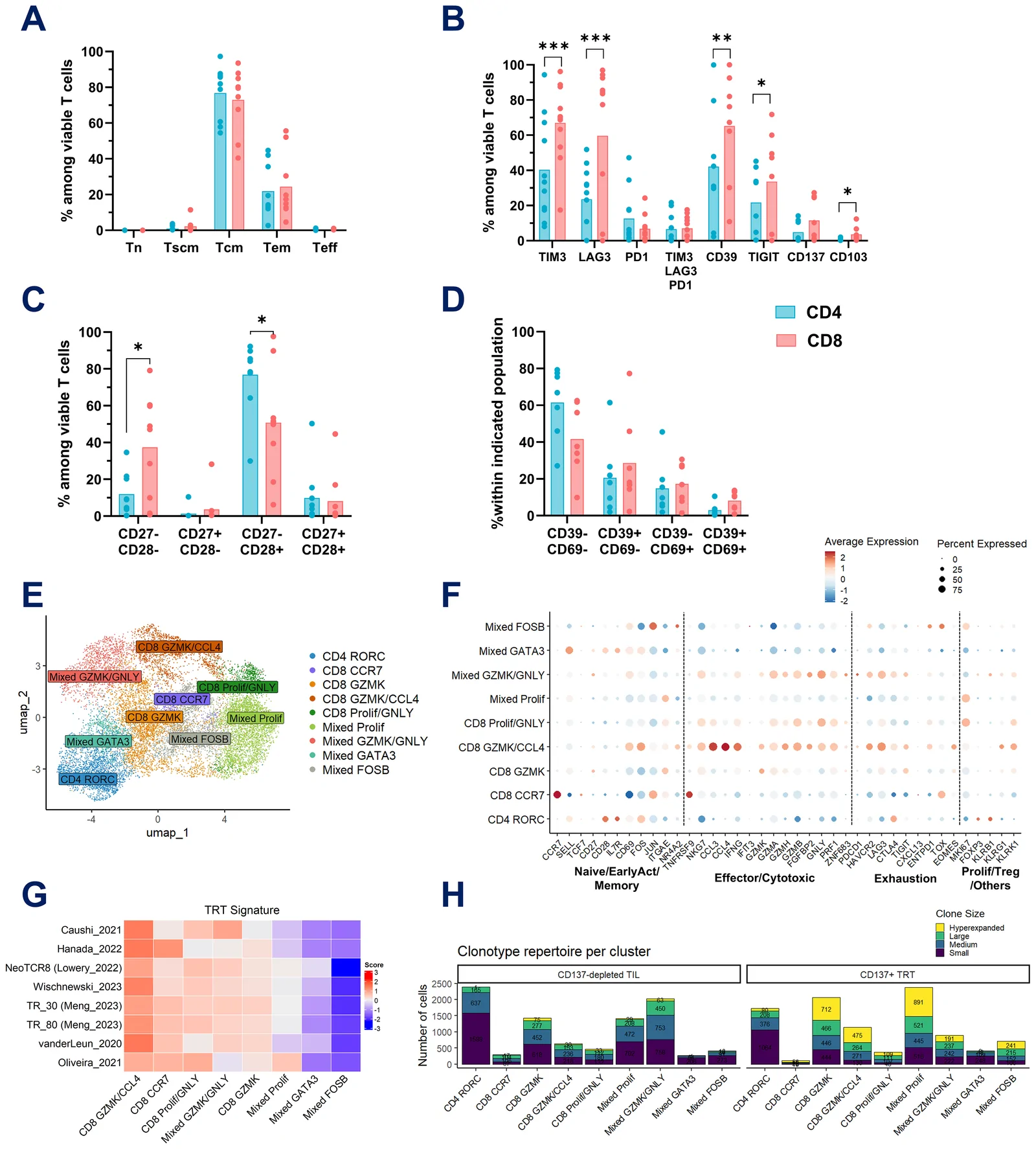
CD137+ TRT Products Maintain Favorable Phenotypic and Transcriptional Features Associated with Therapeutic Potential. **(A-D)** GBM-TIL products manufactured using the automated TRT Process on the CliniMACS Prodigy were characterized with flow cytometry to assess **(A)** T cell differentiation status: Tn (CD45RA+ CD62L+ CD95-), Tscm (CD45RA+ CD62L+ CD95+), Tcm (CD45RA- CD62L+), Tem (CD45RA- CD62L-), and Teff (CD45RA+ CD62L-); **(B)** activation and exhaustion marker expression; **(C)** CD27 and CD28 co-expression, and **(D)** CD39 and CD69 co-expression. **(E)** UMAP viualization of T cell clusters identified by single-cell RNA sequencing of GBM-TIL products (n=7). **(F)** Gene expression profiles of CD8+ and mixed T cell clusters identified within GBM-TIL products. **(G)** Tumor-reactive T cell (TRT) signature scores of T cell clusters based on eight published TRT gene signatures. **(H)** Relative abundance of TCR clonotypes within clusters between CD137+ TRT and CD137-depleted TIL products grouped according to clonotype abundance (hyperexpanded: <10%, large: 1-10%, medium: 0.1-1%, and small: 0.01-0.1%).

We also determined the frequency of activation and exhaustion phenotypic markers express on CD4 and CD8 T cells in the final cell product. We observed high frequencies of TIM-3, LAG-3, and CD39, and modest frequencies of TIGIT. PD-1, CD103, and CD137 were detected at lower frequencies (**Figure 4B**). CD137 is transiently upregulated following activation, returning to baseline at approximately 48 hours after stimulation and therefore does not remain elevated throughout culture (24). Notably, TIM-3, LAG-3, TIGIT, CD39 and CD103 were more frequently expressed on CD8 T cells relative to CD4 T cells (**Figure 4B**). Very few triple positive (LAG-3+/TIM-3+/PD-1+) T cells were detected, a phenotype associated with terminally exhausted T cells (**Figure 4B**). The expansion methodology also influenced the expression profile of exhaustion markers, as CD137+ TRTs generated on the CliniMACS Prodigy displayed lower expression of exhaustion markers compared with products generated with the small scale, bench-top methodology, particularly PD1, LAG3, and TIGIT (**Supplementary Figure S5B**). The majority of T cells within the final product also displayed favorable surface marker characteristics previously associated with responses to TIL therapy, including high frequency of CD28+ T cells and a high frequency of CD39/CD69 double negative T cells (**Figures 4C, D**) (35–38).

To further characterize the final CD137+ TRT products generated using the CliniMACS Prodigy TRT Process, we performed single-cell RNA sequencing on products from seven patients, yielding transcriptomic profiles for 14,913 T cells. Unsupervised clustering resulted in one CD4, four CD8, and four T cell clusters containing both CD4 and CD8 T cells (**Figure 4E**, **Supplementary Figures S5C, D**). The CD4 cluster expressed markers associated with less differentiated T cell states (*SELL* encoding CD62L, *CD28*, *IL7R*), as well as genes associated with Th17 cells (*RORC*, *CCR6*, *KLRB1*) (**Supplementary Figure S5E**). We identified four distinct CD8 TIL clusters expressing different expression patterns of granzymes (*GZMK*, *GZMH*, *GZMB*), effector molecules (*FGFBP2*, *GNLY*, *PRF1*, *IFNG*), cell division-related genes (*MKI67*, *TOP2A*, *TUBB*), and exhaustion genes (*HAVCR2* encoding *TIM3*, *LAG3*, *TIGIT*, *ENTPD1*, *TOX*) (**Figure 4F** and **Supplementary Figures S5F, G**). Notably, one of these CD8 TIL clusters exhibited high expression of *CCL3* and *CCL4*, ligands for CCR5 implicated in immune recruitment. This CD8 GZMK/CCL4 cluster displayed a predominant cytotoxic and exhaustion transcriptional profile and was highly scored across the eight public tumor reactivity signatures previously used to evaluate the starting TIL populations prior to processing (**Figure 4G**). In addition to CD137+ TRT products, we also analyzed the transcriptomes of 11,556 CD137-depleted TILs generated in parallel from six of the same patients. Analysis of the TCR repertoire revealed that the proportion of T cells that harbor hyperexpanded TCRs was greater in CD137+ TRT products compared to matched CD137-depleted TIL products (**Figure 4H**).

In summary, CD137+ TRTs expanded using the CliniMACS TRT Process generated CD4+ and CD8+ TIL products enriched for activated, clonally expanded TIL populations while maintaining differentiation states and transcriptional profiles associated with tumor-reactivity.

### 2.5 Manufactured GBM CD137+ TRTs demonstrate potent cytotoxicity against autologous tumor cells

The functional evaluation of TIL products remains challenging due to their polyclonal nature and the diverse, often undefined, antigen specificities that contribute to tumor recognition. To overcome this limitation, we generated autologous tumor cell lines from each tumor digest to directly assess the tumor-reactive capacity of TRTs generated from GBM UA. Primary GBM cell lines were successfully established from patient samples with high efficiency (**Supplementary Figure S6A**).

Dissociated UA and subsequently established primary cell lines were analyzed for expression of selected tumor-associated antigens (TAAs), as well as MHC-I. In line with previous reports (10, 11), we observed heterogeneous expression of tumor antigens in dissociated UA samples, with on average 39% cells expressing GD2, 33% expressing GLAST, 23% expressing B7-H3 and IL13Rα2, and roughly 7% expressing HER2 (**Supplementary Figure S6B**). As expected, TAA expression became more frequent and uniform in established cell lines relative to the initial UA digest, where 87% of the cells expressed B7-H3, 75% GD2, 48% GLAST, 23% IL13Rα2, and 21% were HER2 positive (**Supplementary Figure S6C**).

The majority of tumor cells within the GBM aspirates were MHC-I+, and MHC-I expression levels were notably higher than those observed in CD45+ cells (**Supplementary Figure S6D**). In addition, the frequency of MHC-I expression within primary cell line further increased from 75% to 98% of viable cells (**Supplementary Figure S6E**). We further confirmed that IFN-γ sensing machinery remained intact, as MHC-I expression was upregulated by exogenous IFN-γ addition (**Supplementary Figure S6F**). These autologous GBM cell lines also established tumors following orthotopically injected into murine brains (**Supplementary Figure S6G**).

We next sought to determine the impact of CD137 enrichment on the functional activity of TRTs generated by the CliniMACS Prodigy. From each digested surgical aspirate, we generated, CD137+ TRT products in parallel with unenriched TIL and CD137-depleted TIL products (**Figure 5A**). Co-culture of CD137+ TRT products demonstrated more potent cytolytic activity against autologous tumor cells compared to unenriched TILs or CD137-depleted TILs from the same patient (**Figure 5B**, **Supplementary Figure S7**). Interestingly, we observed no difference in the cytotoxic activity of the CD137+ TRT products derived from patients receiving low or high dose dexamethasone (**Supplementary Figure S8**). CD137+ TRT products exhibited increased perforin, granzyme and cytokine production following co-culture with autologous tumor cell lines compared with unenriched TIL or CD137-depleted TIL products (**Figure 5B**, **Supplementary Figure S9**). CD137+ TRT products manufactured in the CliniMACS Prodigy using the TRT Process were consistently and significantly more potent than unenriched TIL products at killing autologous tumor lines (n=10) (**Figure 5C**). Cytotoxicity of CD137+ TRT products against allogeneic patient-derived GBM cell lines was markedly reduced, supporting the tumor specificity of the response toward autologous tumor cells (**Figure 5D**). Because the CD137+ enrichment process is antigen agnostic, we assessed the presence of additional potential reactivities in the CD137+ TRT products. We stimulated CD137+ TRT, unenriched TIL or CD137-depleted TIL products with PepTivators containing peptides from common viruses (cytomegalovirus (CMV), Epstein-Barr virus (EBV), Influenza antigens) or cancer associated antigens reported to be expressed in GBM (NY-ESO1, survivin and PRAME) (39–42). CD137+ TRT products demonstrated reduced frequencies of CMV-, EBV- and influenza peptide-induced intracellular cytokine expression compared with unenriched TIL, or CD137-depleted TIL products, suggesting reduced representation of irrelevant anti-viral reactivities in the final CD137+ TRT product (**Figure 5E**). Conversely, CD137+ TRT products were enriched for reactivity against shared cancer antigens, suggesting the enrichment of cancer-related reactivities by the CD137+ enrichment and TRT Process (**Figure 5F**). Collectively, these findings demonstrate the therapeutic potential of CD137+ TRT products in GBM immunotherapy and support the use of the automated CliniMACS TRT Prodigy Process for the efficient manufacturing of tumor-reactive TILs for GBM.

**Figure 5:**
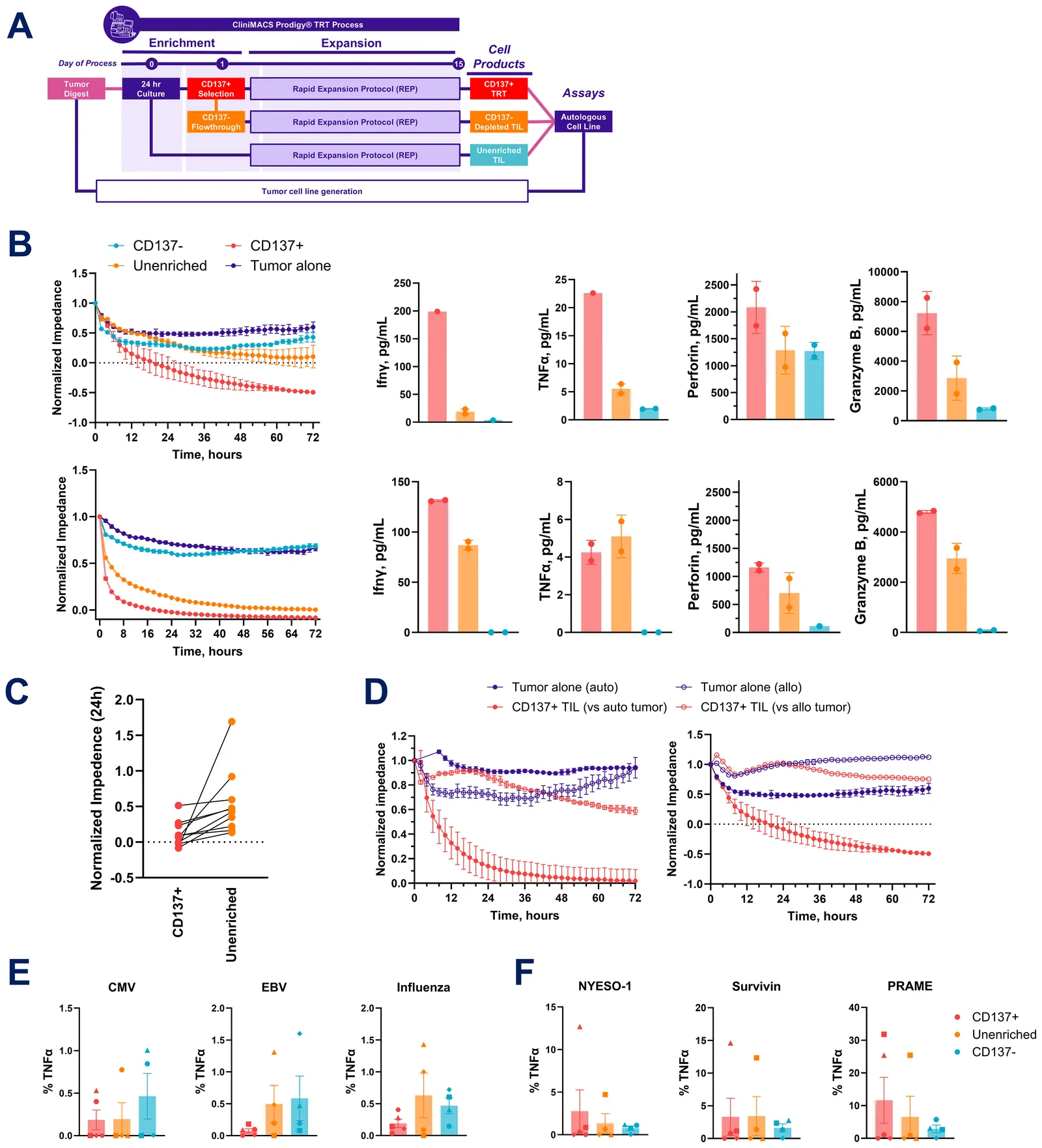
Manufactured GBM CD137+ TRTs demonstrate potent cytotoxicity against autologous tumor cells. **(A)** Schematic representation of the workflow used to generate matched CD137+ TRT, CD137-depleted and unenriched TIL. **(B)** Autologous tumor cells lines were either cultured alone or in presence of CD137+ TRTs, unenriched TILs or CD137-depleted TILs for 72 hours. Growth/killing was assessed with impedance-based XCELLigence system. Decreasing impedance indicates reduction of the viable tumor cell number. Cytokine concentration in coculture supernatants was evaluated with MACSPlex assays. Representative patient products. E:T=8:1 (upper plot) and E:T=2:1 (lower plot). Right panels: cytokines assessed at 72 hours from supernatants from left panel. **(C)** Summary of 24h cytotoxicity from all donors measured. **(D)** Autologous (closed circles) and allogeneic tumor cells (open circles) were cultured alone (purple) or co-incubated with the CD137+ TRT products for 72 hours. Cytotoxicity was assessed as in **(B)**. Two representative patient products are shown. **(E, F)** CD137+ TRT products were stimulated with indicated PepTivators for 24h and the reactivity was assessed by intracellular cytokine staining. Frequencies of TNFa-secreting cells are shown upon stimulation with viral **(E)** and common tumor antigen **(F)** PepTivators. Values were normalized by subtracting the background percentage of positive cells in the unstimulated condition.

## 3 Discussion

GBM is characterized by poor clinical outcomes and a substantial unmet need for effective therapies. However, the limited immune infiltration characteristic of GBM has historically presented a major barrier to TIL manufacturing, with insufficient starting T cell numbers limiting broader clinical translation (43, 44). In this study, we demonstrate the feasibility of generating clinically relevant TIL products from GBM tumor material using a closed, automated manufacturing process incorporating CD137-based enrichment of tumor-reactive T cells. These findings provide a framework for overcoming key limitations that have restricted the application of TIL therapy to immunologically cold tumors.

We first established UA-derived tumor material as a practical source for TIL manufacturing, demonstrating recovery of sufficient T cells from GBM specimens despite the low immune infiltration characteristic of this disease. A subset of these T cells expressed CD137 and displayed phenotypic and transcriptional features associated with tumor-reactive populations, including clonal expansion and enrichment of published T cell reactivity signatures. Although clinical experience with GBM TIL therapy remains limited, studies across other tumor indications have identified several product characteristics associated with improved therapeutic benefit, including CD8+ T cell frequency, CD28 expression, CD39/CD69 phenotype, and favorable differentiation states (35, 36, 38). The CD137+ TRT products generated in this study retained many of these characteristics, including favorable memory profiles, limited terminal exhaustion phenotypes, and enrichment of clonally expanded T cell populations. Importantly, tumor-reactive potential was supported by transcriptomic signatures associated with reactive T cell states, while functional assays demonstrated enhanced cytotoxicity against autologous GBM tumor cells. While these findings demonstrate manufacturing feasibility and ex vivo antitumor activity, clinical evaluation will be required to determine whether CD137-enriched TRT products translate into durable therapeutic responses in patients with GBM.

Although this study focused on ultrasonic aspirates as the starting material for TIL manufacturing, the TRT Process is not inherently restricted to UA-derived material and is compatible with other dissociated tumor specimens, as demonstrated previously across multiple solid tumor types (9). In our experience, ultrasonic aspirates typically provide a readily available and larger source of tumor material than surgically selected tumor fragments, facilitating recovery of sufficient T cells for manufacturing. Previous studies comparing UA-derived material with matched resected tumor specimens have demonstrated comparable immune cell composition, viability, and functional characteristics, supporting UA as a representative source of GBM tissue for TIL-based applications (21). While lower-input tumor specimens may present practical challenges for the current magnetic enrichment workflow, rather than the biological applicability of the approach itself, strategies to further extend the platform to limited tissue samples remain an active area of investigation. Whether differences in tissue source, including variations in tumor regions sampled, processing methods, or cellular composition, ultimately influence TIL yield, phenotype, or functional activity remains to be determined.

A potential concern in applying CD137-based selection to GBM is the enrichment of regulatory T cells, as GBM tumors are known to contain substantial immunosuppressive immune populations. Although CD137 expression was observed on Tregs within the starting tumor material, these cells did not preferentially expand during the REP and represented only a minor population within the final CD137+ TRT products. This observation is consistent with a previous TIL study in melanoma, where Treg frequencies were reduced during manufacturing relative to the starting tumor digest and remained at low in final products, including products associated with clinical response. In those studies, Tregs represented approximately 1% of final products from TIL therapy responders suggesting that the low frequency of Tregs observed within the CD137+ TRT products is within the range observed in clinically relevant TIL products (35). Together, these findings suggest that the combination of CD137-based selection and the TRT Process does not promote preferential expansion of regulatory populations, despite their presence in the tumor digest.

The impact of preoperative patient treatment on TIL procurement and manufacturing is an important consideration for future clinical translation. In this study, higher-dose dexamethasone treatment (>8 mg/day) was associated with reduced T cell recovery, whereas lower doses did not significantly affect TIL yield, suggesting that TIL manufacturing remains feasible in the context of clinically necessary corticosteroid use (45, 46). Strategies that reduce corticosteroid exposure, including dexamethasone-sparing approaches such as bevacizumab, may therefore improve the availability and quality of starting material for cellular therapy approaches (47, 48). These observations highlight that optimization of the tumor microenvironment before collection may represent an important component of future TIL manufacturing strategies. For example, the use of immune-modulating interventions designed to increase tumor immune infiltration prior to surgery, such as neoadjuvant immune checkpoint blockade, represents a potential avenue to further improve TIL yield and therapeutic potency, particularly in poorly infiltrated tumors (49). However, the optimal approach for modulating the tumor microenvironment before TIL collection remains to be determined and care should be taken to interrogate if such interventions may also increase the frequency of suppressive Tregs or MDSCs.

Our study suggests that tumors that have been historically overlooked for TIL therapy due to low mutational burden, limited immune infiltration, or high frequencies of non-tumor-reactive T cells may still harbor rare, tumor-reactive T cell populations that can be recovered and expanded through targeted selection strategies. Thus, enrichment for markers of tumor reactivity, such as CD137, combined with automated and standardized manufacturing workflows, may improve the consistency and scalability of TIL production in settings where conventional bulk expansion is insufficient. This approach provides a framework for extending the TIL therapeutic landscape beyond traditionally responsive tumor types and toward immunologically challenging cancers with substantial unmet clinical need.

## 4 Materials and Methods

### 4.1 Ultrasonic surgical aspiration

Tumor ultrasonic aspirates were collected from patients with histologically confirmed glioblastoma multiforme (GBM) according to the WHO 2021 classification. Material collection was performed with informed patient consent following approval by the institutional review boards of the University Hospital Essen (21-10475-BO), University Hospital Bonn (501/21), and University Hospital Frankfurt (2022-1042), in accordance with institutional and national regulations.

During surgical resection, tumor material was collected using a cavitronic ultrasonic surgical aspirator (CUSA). Aspirated tissue was retained in the collection bag, stored at 4 °C, transported to the laboratory (typically on the day of surgery), and processed as described below.

### 4.2 GBM aspirate dissociation

Upon arrival, ultrasonic aspirates were transferred to conical tubes, pelleted, washed with PBS, and subjected to red blood cell lysis using ACK lysis buffer (Gibco). Where immediate processing was not possible, samples were stored overnight at 4 °C in Cell Storage Solution (Miltenyi Biotec) supplemented with 1× Antibiotic-Antimycotic (Gibco). Samples were subsequently dissociated using the Tumor Dissociation Kit, human (Miltenyi Biotec), on a gentleMACS Octo Dissociator (Miltenyi Biotec) with Heaters according to the manufacturer’s instructions. Digested material was washed and sequentially filtered through 1000 μm, 100 μm, and 70 μm cell strainers. In some cases, additional debris removal procedure was performed with debris removal solution (Miltenyi Biotec).

### 4.3 Flow cytometry

GBM tumor digests, expanded TIL products, and primary GBM cell lines were analyzed by multicolor flow cytometry using antibody panels designed to characterize immune composition, tumor-associated antigen expression, T cell differentiation, and activation/exhaustion phenotypes (**Supplementary Figures S1B, C** and **S2A, B**). Cells were stained in PEB buffer (PBS containing 2 mM EDTA and 0.5% human serum albumin; Miltenyi Biotec) with antibody cocktails for 10 min at 4 °C. Where indicated, intracellular staining was performed using Inside Fix and Inside Perm reagents (Miltenyi Biotec) according to the manufacturer’s instructions. Samples were acquired on MACSQuant flow cytometers (Miltenyi Biotec) and analyzed using MACSQuantify software and GraphPad Prism (version 10.1.2). A description of all the antibodies used in the study can be found in **Supplementary Table 3**.

### 4.4 Automated TRT manufacturing

Automated TIL manufacturing was performed using the CliniMACS Prodigy^®^ Tumor-Reactive T cell (TRT) Process as previously described (9). At the start of the process, on day 0, fresh or cryopreserved GBM tumor digests were loaded into a primed TS520 Tubing Set and cultured overnight in TexMACS GMP Medium supplemented with 3% heat-inactivated human AB serum (Capricorn), 1% penicillin-streptomycin (Gibco), and 50 ng/mL each of IL-7 and IL-15 (Miltenyi Biotec).

On day 1, following overnight culture, cells were automatically labeled with CliniMACS^®^ CD137 GMP Biotin and CliniMACS^®^ Anti-Biotin GMP MicroBeads before magnetic enrichment within the CliniMACS Prodigy. CD137-enriched cells were automatically transferred into the Centricult chamber and expanded in TexMACS GMP Medium (3% AB serum 1% Penicillin-Streptomycin, 50 ng/mL IL-7, IL-15 and IL-21) in presence of 4x10^8^ irradiated feeder cells and 30 ng/mL MACS^®^ GMP CD3 pure (Miltenyi Biotec).

The negative fraction, obtained following CD137 enrichment and containing the CD137 depleted tumor digest, was transferred to a second CliniMACS Prodigy for parallel expansion. Additionally, an aliquot of the culture prior to enrichment was similarly expanded in parallel on a third CliniMACS Prodigy to generate an unenriched TIL product.

After magnetic selection cells were cultured for an additional 14 days according to the standard TRT Process activity matrix. Beginning on process day 6, cultures were monitored by assessment of glucose and lactate concentrations and viable cell counts. Media exchanges were adjusted when required to maintain optimal lactate and glucose thresholds. On process day 15, the cell products were automatically rebuffered with CliniMACS Final Formulation Solution (Miltenyi Biotec) and transferred into a target cell bag for harvest and analysis.

### 4.5 Small-scale tumor-reactive T cell generation

For manual, benchtop-scale TRT generation, dissociated GBM tumor digests containing both immune and tumor cells followed a protocol which generally mirrored the timing and steps of the automated process. On day 0, tumor digests were cultured overnight at 37 °C and 5% CO_2_ in TexMACS Medium supplemented with 5% human AB serum, 1× Gibco™Antibiotic-Antimycotic (Thermo Fisher Scientific), and 50 ng/mL each of IL-7 and IL-15. On process day 1, CD137+ cells were enriched using CD137 MicroBeads and MACS separation columns according to the manufacturer’s instructions (Miltenyi Biotec; Cat. No. 130-042-401). Approximately 1x10^4^ digests from CD137-enriched, CD137-depleted, and unenriched fractions were entered into a rapid expansion protocol (REP) for 14 days in the presence of 30 ng/mL anti-CD3 antibody (OKT3; Miltenyi Biotec), irradiated feeder cells, and TexMACS Medium supplemented with 3% human AB serum, 1% Penicillin-Streptomycin, and 50 ng/mL each of IL-7, IL-15, and IL-21. Starting on day 6 of the process, cells were monitored for density and cultured to maintain expansion and viability. Generally, feeding and replacement of media began on day 6 of the process and continued until the end of the culture time. On day 15 of process, the cells were harvested for analysis.

### 4.6 Tumor cell line generation and validation

Autologous GBM cell lines were established by plating aliquots of tumor digests onto laminin-coated six-well plates (MACSMatrix; Miltenyi Biotec) in DMEM/F12 supplemented with 1% Antibiotic-Antimycotic (Thermo Fisher Scientific), 2% B27 supplement (Gibco), 20 ng/mL human EGF, 20 ng/mL human FGF (Thermo Fisher Scientific), and 5 μg/mL human heparin (Sigma-Aldrich). Where indicated, cells were transduced with lentiviral vectors expressing GFP and luciferase and selected using puromycin. To verify tumorigenicity, 1,000 cells were suspended in 2 μL of PBS and injected intracranially into 3-day-old pups generated from breeding male and female NOD SCID IL2 receptor gamma knockout (NOG; Taconic) mice using 0.3 mL BD Micro-Fine insulin syringes. Prior to injection, the skin over the injection site was treated with topical EMLA anesthetic cream. Cells were injected into the left lateral ventricle (approximately 1 mm caudal and 1 mm lateral to bregma). Mice were monitored by *in vivo* bioluminescence imaging (IVIS) and sacrificed 3–6 months after implantation.

### 4.7 Functional assays

T cell functionality was assessed using co-culture assays with autologous primary GBM cell lines. Briefly, 5x10^3^ to 1x10^4^ primary patient GBM cells were seeded into laminin-coated 96-well plates and allowed to adhere prior to the addition of effector cells. Effector cells were added at indicated effector-to-target ratios. Tumor cell growth kinetics were analyzed using either live-cell imaging system Incucyte S3 (Sartorius), or xCELLigence RTCA MP analyzer (Agilent Technologies, Santa Clara, California, USA) according to the manufacturers’ instructions. For impedance-based assays, baseline impedance was recorded for each sample prior to the addition of effector cells and all subsequent measurements were normalized to this baseline.

For cytokine analysis, supernatants were collected following co-culture assays, centrifuged to eliminate residual cells and debris, and snap-frozen. Supernatant was thawed and analyzed with the Cytotoxic T/NK Cell MACSPlex Cytokine Kit (Miltenyi Biotec) according to manufacturer’s instructions.

### 4.8 Single-cell RNA sequencing

TIL were enriched from tumor digests using the REAlease^®^ CD4/CD8 (TIL) MicroBead Kit, human (Miltenyi Biotec) prior to the single-cell preparation. Expanded TRT products were analyzed without additional enrichment. Following washing in RPMI supplemented with 10% FBS, cell viability and number were determined in an automated cell counter. Cell suspensions targeting the recovery of approximately 10,000 cells were loaded into the Chromium X/iX instrument (10X Genomics) for single-cell partitioning. Single-cell cDNA libraries were prepared using Chromium Next GEM Single Cell 5’ Reagent Kit v2 (10X Genomics) according to the manufacturer’s instructions.

Libraries were quantified by Qubit dsDNA HS Assay Kit (ThermoFischer Scientific), Bioanalyzer High Sensitivity DNA Assay (Aligent) and quantitative PCR using the NEG Next Library Quant Kit for Illumina (New England Biolabs). Sequencing was performed on either the Illumina NextSeq 550 or NextSeq 2000 sequencer, with sufficient sequencing depth to generate at least 10,000 paired-end reads per cell for VDJ libraries and 30,000 pair-end reads per cell for gene expression libraries.

Sequenced data were pre-processed with CellRanger Software v. 7.1.0 (10X Genomics) using the *mkfastq*, *count* and *multi* pipelines. The following reference datasets available from 10X Genomics were used for the analysis: refdata-gex-GRCh38-2020-A and refdata-cellranger-vdj-GRCh38-alts-ensembl-7.1.0. Processed data was further analyzed using a custom R pipeline (R version 4.4.3) that incorporated Seurat (version 5.2.1) (50) and scRepertoire (version 2.3.4) (51) R packages for data processing and visualization. Quality control was performed independently for each sample. Low-quality cells and empty droplets were removed by applying minimum thresholds for the number of detected genes and molecules per cell. Cells with a high proportion of mitochondrial gene expression, indicative of poor cell quality, were excluded. Multiplets were identified using the R package scDblFinder (version 1.20.2) (52) and removed, together with cells exceeding the maximum thresholds for detected genes and molecules per cell. To further eliminate contaminating non-T cell populations, each sample was processed using the Seurat workflow, including SCTransform normalization, principal component analysis (PCA), and cell clustering. Clusters corresponding to non-T cell subsets were excluded from subsequent analyses. The filtered datasets from all samples were subsequently merged for downstream analyses, including data visualization.

### 4.9 Statistical analysis

Statistical analyses were performed using GraphPad Prism (version 10.1.2). Comparisons between paired groups were performed using paired two-tailed Student’s t-tests, whereas comparisons involving more than two matched groups were analyzed using one-way repeated-measures ANOVA with appropriate *post hoc* multiple comparison testing. Statistical tests are indicated in the corresponding figure legends. Description of p values <0.05 (*), <0.01 (**) and <0.001 (***) are indicated where appropriate.

## Data Availability

All single cell and RNA sequencing data has been deposited here: (https://www.ncbi.nlm.nih.gov/geo/query/acc.cgi?acc=GSE349606). Additional data is available upon request from the corresponding author(s).
